# Understanding cross-talk–induced anode slippage in high-voltage mid-Ni NCM/graphite full cells

**DOI:** 10.1080/14686996.2025.2502324

**Published:** 2025-05-16

**Authors:** Seungjae Suk, Namgyu Yoo, Youngsu Lee, Jaesub Kwon, Heeju Ahn, Seungsu Yoo, Jaewoon Lee, Haneul Kim, Joongho Bae, Jongwoo Kim, Chiho Jo, Yong-Tae Kim, Kyu-Young Park

**Affiliations:** a Department of Materials Science and Engineering, Pohang University of Science and Technology (POSTECH), Pohang, Republic of Korea; b Graduate Institute of Ferrous & Eco Materials Technology (GIFT), Pohang University of Science and Technology University, Pohang, Republic of Korea; c LG Energy Solution, Research Park, Daejeon, Republic of Korea

**Keywords:** Lithium-ion batteries, anode slippage, high-voltage cycling, single-crystal mid-Ni NCM, full-cell, cross-talk

## Abstract

While high-voltage operation of mid-Ni layered oxide cathodes in full-cell Li-ion batteries is essential for achieving high energy density, it inevitably accelerates electrode degradation, ultimately resulting in capacity loss. However, the underlying degradation mechanisms under high-voltage conditions remain poorly understood. In this study, we reveal that anode slippage – induced by cross-talk-driven surface degradation – is the dominant factor in capacity fade during high-voltage (4.35 or 4.40 V) cycling of single-crystal mid-Ni layered oxide (SC-NCM)/graphite pouch full-cells. Electrochemical and post-mortem analyses show that, although high-voltage operation induces cathode surface degradation, including lattice oxygen loss and phase transitions, its direct impact on capacity loss is relatively minor compared to that of the anode. Instead, anode degradation is primarily caused by cross-talk effects from cathode Ni dissolution, which promote the accumulation of irreversible organic byproducts – such as LiO*x* and Li_2_CO_3_ – within the solid electrolyte interphase (SEI) layer of the graphite anode. This leads to increased resistance and reduced anode electrochemical activity, disrupting electrode balance and accelerating full-cell capacity fade. These findings highlight the critical role of anode degradation in high-voltage operation and emphasize the importance of mitigating cross-talk effects. A comprehensive understanding of cross-talk–induced anode slippage is therefore critical for the rational design of high-voltage mid-Ni full-cell systems with long-term durability.

## IMPACT STATEMENT

This study identifies cross-talk – induced anode slippage as the dominant degradation mechanism in high-voltage mid-Ni NCM/graphite full-cells, emphasizing the need for mechanistic understanding for rational design of durable high-voltage systems.

## GRAPHICAL ABSTRACT

**Figure:**
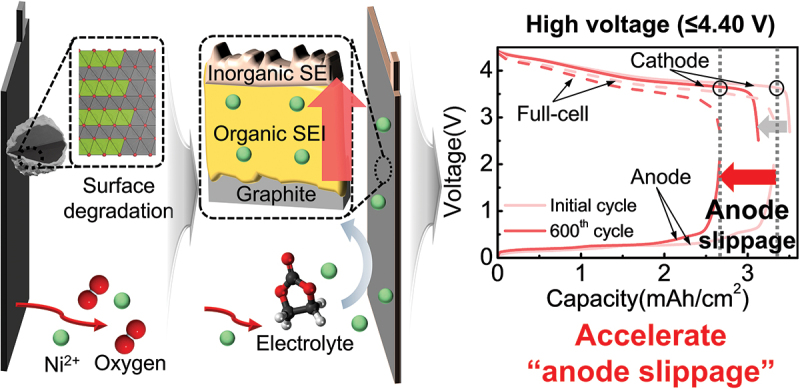

## Introduction

Over the past two decades, lithium-ion battery (LIB) technology has advanced rapidly in parallel with the growth of the electric vehicle (EV) market [1]. Among various battery design parameters, the selection of cathode material chemistry has had a decisive impact on key EV performance metrics such as driving range per charge and cost per battery pack [2]. In line with this need, high-Ni layered oxide cathodes (Ni ≥ 80%, high-Ni NCM) have received significant attention due to their superior energy density, which supports the demand for extended driving range. However, high-Ni NCMs exhibit inferior cycle life due to anisotropic lattice changes during lithium insertion/extraction, which promotes crack propagation along grain boundaries within particles [3], leading to the formation of rock-salt phases [4] and electrolyte decomposition [5] on newly exposed surfaces. Furthermore, these cathode materials undergo thermal decomposition and oxygen release at relatively low temperatures (~200 °C), increasing their vulnerability to thermal runaway [6,7]. These inherent limitations remain key barriers to the commercialization of high-Ni NCM cathodes. While lithium iron phosphate (LFP) cathodes have recently emerged as viable alternatives for cost-effective battery design that mitigate these issues [1,8,9], they still present distinct challenges, including lower energy density [10], poor low-temperature performance [11], and potential supply chain vulnerabilities due to geographically concentrated production capacity [12].

Single-crystal lithium nickel cobalt manganese oxide (SC-LiNi*_x_*Co*_y_*Mn_1−*x*−*y*_O_2_, 0.4 ≤ x ≤ 0.6; hereafter SC mid-Ni NCM) cathodes with medium Ni content (<60%) have emerged as a promising alternative for moderate-range EVs, offering improved cost flexibility through reduced nickel content compared to high-Ni NCMs [13–15]. Notably, SC mid-Ni NCM features lower surface nickel concentration and reduced surface area, substantially limiting electrolyte side reactions compared to high-Ni NCM [16,17] while simultaneously providing superior thermal stability [18]. These properties enable the implementation of space-efficient cell-to-pack architectures despite their layered oxide chemistry, presenting opportunities for substantial cost reduction at the battery pack level and meaningful improvements in practical energy density parameters [19]. Furthermore, SC mid-Ni NCM exhibits operational reliability across a more extensive temperature range than LFP alternatives [20], factors that have collectively positioned this material as an increasingly prominent candidate for long-lifecycle EV battery applications.

To meet the performance demands for such applications, recent guidelines from the US Council for Automotive Research indicate that SC mid-Ni NCM materials must achieve a pack-level energy density of approximately 235 Wh/kg or higher to remain competitive with internal combustion engine vehicles in the moderate-range EV segment [21]. This target corresponds to roughly 350 Wh/kg at the cell level and requires a minimum of 800 Wh/kg at the cathode material level. For SC-NCM compositions containing 60% nickel, achieving this level of material energy density necessitates operation at elevated upper cut-off voltages of 4.45 V *vs*. Li/Li⁺ (equivalent to 4.40 V in NCM/graphite full-cell configurations), enabling the extraction of a specific capacity of approximately 200 mAh/g.

However, operating at such high voltages introduces critical degradation challenges, particularly for Ni-based layered oxide cathodes. Previous investigations have reported that Ni-based layered oxide cathodes experience complex degradation mechanisms encompassing both mechanical and chemical phenomena during high-voltage operation. Pronounced electrochemical creep and significant lattice strain  [22,23] can initiate mechanical fractures and disrupt electrical contact at the electrode–current collector interface. High-voltage cycling also promotes the formation of stacking faults, which can lead to bending and distortion of the cathode microstructure [24]. Concurrently, chemical degradation – particularly surface oxygen release – not only facilitates transition metal dissolution [25] but also reinforces mechanical breakdown through positive feedback mechanisms [24], further accelerating performance decay during prolonged cycling.

Importantly, such degradation is not confined to the cathode alone but extends to the entire full-cell system. For example, in high-Ni layered oxide/graphite full-cells, cross-talk phenomena have been widely reported, where transition metal ions dissolved from the cathode migrate and induce degradation at the anode [26]. Under high-voltage and high-temperature conditions [27], Ni ions are known to deposit on the anode surface, thickening the SEI layer [28], and catalyzing electrolyte decomposition [29]. Such phenomena can be exacerbated under fast charging conditions, which presents a significant challenge for implementing mid-Ni NCM cathodes paired with graphite anodes in electric vehicle full-cell systems [30,31]. Furthermore, parasitic reactions at the positive electrodes progressively alter the capacity balance between electrodes from their initial alignment, a mechanism known as electrode slippage [32]. While such degradation pathways have been increasingly characterized in high-Ni systems, the interplay between cathode and anode degradation under high-voltage operation in SC mid-Ni NCM/graphite full-cells remains poorly understood. In particular, comprehensive understanding of how each electrode contributes to long-term capacity loss and cell imbalance at elevated voltages is still lacking.

In this study, we systematically investigated the degradation mechanisms in high-voltage (≥4.35 V *vs*. graphite) pouch full-cell configurations using single-crystal mid-Ni layered oxide (SC-NCM6)/graphite cells. While cathode degradation – such as oxygen release, surface phase transitions, and internal cracking – was consistently observed and aligned with previous findings, these changes alone could not account for the observed capacity loss in full-cell systems. Instead, our analysis revealed that anode slippage plays a dominant role in capacity fade during long-term cycling. Crucially, this slippage was not an isolated phenomenon but was directly linked to cross-talk effects originating from cathode degradation. Transition metal dissolution from the cathode, particularly Ni ions, migrated to the anode surface, where it promoted the growth of resistive SEI layers rich in LiO*_x_* and Li_2_CO_3_ byproducts. This process significantly increased charge transfer resistance and disrupted the ‘*capacity balance*’ between electrodes – effectively limiting the accessible capacity of the cathode due to the constrained lithium storage at the anode. These findings shift the focus of degradation analysis in mid-Ni full-cell systems from cathode instability alone to the interplay between electrodes, highlighting the critical need to mitigate cross-talk pathways to enable stable high-voltage operation.

## Methods

### Cell specifications and full-cell cycle test conditions

The pouch cell consisted of a SC-NCM6 cathode (~17 mg/cm^2^), a graphite anode, a ceramic-coated separator film and a 1.2 M LiPF_6_ in ethylene carbonate (EC)/ethyl methyl carbonate (EMC)/Diethyl carbonate (DEC) (2:7:1, volume ratio) electrolyte, which was designed for high-voltage long cycling electrochemical performance test. The electrochemical charge/discharge cycling performances of NCM6/graphite pouch full-cells were studied via 0.33 C constant current (CC) charge/discharge cycling on Wonik PNE SC model battery testing system in cell voltage ranges from 2.5–4.35 V and 2.5–4.40 V at 45 °C. At the 100^th^ and 300^th^ cycles of the full-cell long-term test, reference performance tests (RPT) were implemented by temporarily stopping the test, allowing the pouch cell to cool, and applying two charge-discharge cycles at 0.3 C with the same voltage conditions at 25 °C. Both the pouch cell fabrication and full-cell long-term cycle tests were performed at LG Energy Solution.

### Post-mortem characterizations

Pouch-type cells were disassembled in an Ar-filled glove box located in a dry room in the discharge state after cycle tests, and the retrieved electrodes were used for half-cell tests. CR2032-type coin cells were assembled with the cycled electrodes and Li metal as a counter electrode and filled with a fresh electrolyte of 1 M LiPF_6_ in EC/EMC/Dimethyl carbonate (DMC) (1:2:1, volume ratio). The electrolyte was selected with a conventional composition known to be stable below 4.50 V (*vs*. Li/Li^+^), as the primary objective was to observe the intrinsic state of the cathode and anode materials. For cycled cathodes, 4.35 V and 4.40 V SC-NCM6 half-cells were cycled in the voltage range 2.5–4.40 V and 2.5–4.45 V (*vs*. Li/Li^+^) at 25°C after 12 h of rest to ensure complete electrolyte wetting. The cell formation conditions include 2 cycles at 0.05 C with CC charge/discharge protocol within the voltage. Afterward, capacity check was conducted at 0.05 C within the same voltage condition. Prior to synchrotron high resolution powder diffraction (HRPD) and soft X-ray absorption spectroscopy (soft XAS) measurements, cathodes were fully lithiated through constant-current discharging at 0.05 C and a subsequent 6-hour voltage hold at 2.5 V. Electrochemical impedance spectroscopy (EIS) was performed over a frequency range from 10^5^ Hz to 1 Hz (ZIVE SP2, WonATech). To minimize changes in charge transfer resistance due to SOC, the cells were fully discharged to 2.5 V *vs*. Li/Li^+^ at 0.1 C in constant current mode followed by constant voltage mode until the C-rate reached 0.01 C.

Synchrotron HRPD characterizations were performed using the 9B beamline at the Pohang Light Source (PLS-II). Measurements were conducted at 25 °C from 15° to 135° in 2θ with 0.008 steps, with a monochromatic X-ray wavelength of 1.5461 Å.

O K-Edge and Ni L-Edge soft-XAS were conducted in 10A2 HR PES-II beamline at the Pohang Light Source (PLS-II). The absorption spectra were recorded in total electron yield (TEY) mode. The energies of the detected elements (Ni, O) were calibrated by the reference sample (NiO). The measured data were processed using the Beagle software [33].

High-resolution transmission electron microscopy images were obtained using a JEOL JEM-2200FS with image spherical aberration corrector at 200 kV, with samples prepared by a focused ion beam (FIB) using a Quanta 3D FEG. The cycled-cathode cross-sectional scanning electron microscopy (SEM) images were also obtained with it.

Time of flight secondary ion mass spectrometry (TOF-SIMS) analysis was performed on an ION-TOF TOF.SIMS 5 spectrometer, equipped with Bi^+^ analysis beam and 0.5 kV Cs sputtering beams for probing secondary-ion fragments. X-ray photoelectron spectroscopy (XPS, K-Alpha+, Thermo Fisher Scientific) was performed using a 200 μm X-ray source and calibrated based on the C 1s peak at 284.5 eV. Graphite anode surface was observed through Field emission SEM (FE-SEM, S-4800, HITACHI). The transition metal content of the separator was quantitatively measured by inductively coupled plasma optical emission spectrometer (ICP-OES Agilent 720, Agilent Technologies Co. Ltd).

## Result and discussion

### Long-term cycling performance of SC-NCM6/graphite full cells at high-voltage

**Figure 1.**
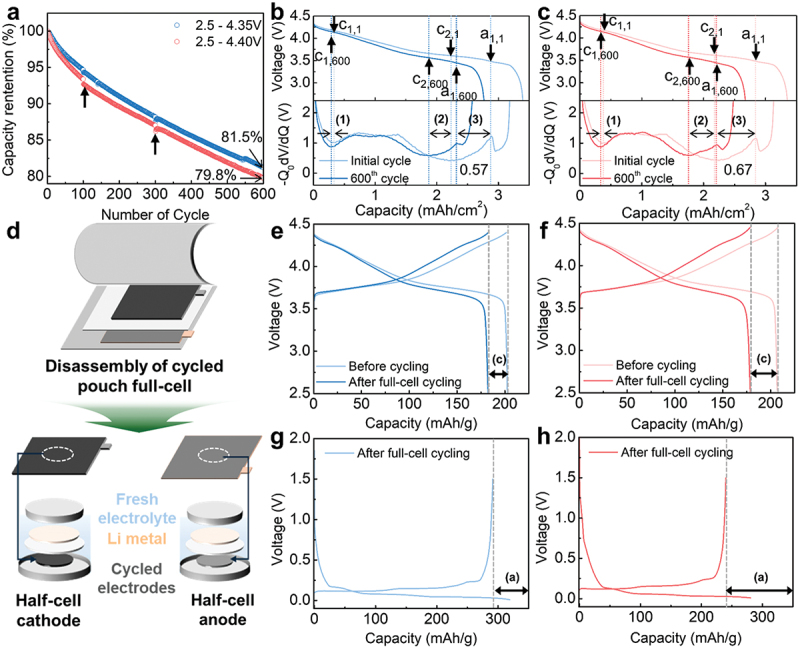
Simultaneous capacity loss of SC-NCM6 cathode and graphite anode in pouch-type full cells during high-voltage long-term cycling. (a) Capacity retention obtained in 2.5–4.35 V(or 4.40 V) at a rate of C/3 after formation cycles. Voltage profiles and differential voltage analysis for (b) 4.35 V and (c) 4.40 V full-cell cycles. (d) Schematic illustration of the post-mortem electrochemical analysis for cycled electrodes; Half-cell charge–discharge curves for the 3rd cycle of SC-NCM6 cathodes retrieved from (e) 4.35 V and (f) 4.40 V full-cells. Half-cell charge–discharge curves for the 1^st^ cycle of graphite anodes retrieved from (g) 4.35 V and (h) 4.40 V full-cells.

Figure 1(a) and Figure S1 illustrate the 600-cycle retention and capacity of SC-NCM6/graphite 40 mAh pouch full-cells when subjected to current application at 0.33 C under upper cut-off voltage conditions of 4.35 V or 4.40 V at 45°C. The kinks indicated by arrows in Figure 1(a) occurred at cycles 100 and 300 due to temporary pause and subsequent resumption of cycling for reference performance test (please refer to the experimental section). At the 4.35 V charging voltage condition, the cell maintained 81.5% capacity retention; however, when the charging voltage was increased to 4.40 V, a rapid decrease in capacity retention was observed from the initial cycling stage, with capacity retention declining below 79.8% after 600 cycles. In terms of discharge capacity, the cell cycled at 4.35 V exhibited an initial value of 198.1 mAh/g, with a capacity decrease of 36.5 mAh/g over 600 cycles. The cell cycled at 4.40 V showed a slightly lower initial discharge capacity of 195.6 mAh/g, despite the expectation of higher de-lithiation at elevated voltages (Figure S1). This observation suggests that degradation may have already initiated during or immediately after the initial formation cycle under the 4.40 V condition, limiting the accessible capacity from the beginning of cycling. After 600 cycles, the total reversible capacity loss at 4.40 V (39.6 mAh/g) exceeded that at 4.35 V. The accelerated degradation of full-cell electrochemical performance under high-voltage operation aligns with the observation of increased swelling during post-electrochemical evaluation visual inspection of full-cells at higher voltages (Figure S2).

To compare the influences of cathode and anode on full-cell capacity degradation according to the upper cut-off window, differential voltage analysis (dV/dQ) was performed on the discharge curves at the initial cycle and 600 cycle points (Figure 1(b,c)). Differential voltage analysis can be mathematically modeled as a linear combination of each cathode and anode half-cell based on the relationship equation below, providing a useful non-destructive method to estimate changes in cathode and anode behavior within the full-cell system [32,34].

(1) $$V_{\mathrm{full}-\mathrm{cell}}=V_{\mathrm{cathode}}-V_{\mathrm{anode}}$$

(2) $$\frac{dV_{\mathrm{full}-\mathrm{cell}}}{dQ}=\frac{dV_{\mathrm{cathode}}}{dQ}-\frac{dV_{\mathrm{anode}}}{dQ}$$

Figure 1(b,c) shows the galvanostatic profiles (top) and -Q_0_dV/dQ plots (bottom) according to capacity per area at the initial cycle and 600^th^ cycle of the full-cell for each upper voltage. The -Q_0_ factor served to normalize the derivatives based on cell discharge capacity. In these -Q_0_dV/dQ plots, peaks predominantly arising from the cathode are denoted as c_a,b_ (a: peak number, b: cycle number), while anode-dominant peaks are labeled as a_a,b_. For reference, the characteristic peak deconvolution for the cathode in Figure 1(b,c) utilized half-cell data from SC-NCM6 (see Figure S3 for detailed explanation). At the beginning of discharge, information about the region where H3-2 phase transition occurs in the cathode (c_1_) appears in the -Q_0_dV/dQ. Peak c_2_ corresponds to the M-H1 phase transition of the cathode, while the final a_1_ corresponds to the phase transition process between stage 1 and stage 4 of the anode [32,34].

Through dV/dQ plot analysis, it is suggested that electrode slippage of the anode is the direct cause of reversible capacity reduction in the full-cell, and that higher charging voltages lead to greater electrode slippage. Electrode slippage refers to the phenomenon where parasitic side reactions occurring at the anode and/or cathode interfaces progressively alter the capacity balance between electrodes from their initial aligned state [32,34,35]. Changes in c_1_, c_2_, and a_1_ peak shifts in -Q_0_dV/dQ after 600^th^ cycle compared to the first cycle of the full-cell appear due to reversible capacity loss of each electrode or electrode slippage phenomena [35]. The leftward shift of a_1_(a_1,1_→ a_1,600_, (3)) indicates the degree of anode slippage, while the movements of c_2_(c_2,1_→ c_2,600_, (2)) and c_1_(c_1,1_→ c_1,600_, (1)) are combined results of cathode reversible capacity reduction and anode slippage. Regarding the shift of c_1_((1)), both 4.35 V (Figure 1(b)) and 4.40 V (Figure 1(c)) full-cells slightly shifted to the left by 0.03 mAh/cm^2^ Meanwhile, the change in spacing between c_1_ and c_2_, which can infer the degree of cathode degradation after cycling, was greater at high voltage, with 0.35 mAh/cm^2^ at 4.35 V and 0.41 mAh/cm^2^ at 4.40 V. This indirectly confirms that cathode degradation intensifies under high-voltage operation. Analysis of anode slippage ((3)) reveals values of 0.57 mAh/cm^2^ in the 4.35 V full-cell (Figure 1(b)) compared to 0.67 mAh/cm^2^ in the 4.40 V system (Figure 1(c)), demonstrating that higher charging voltages substantially accelerate slippage at the anode. This comparison clearly indicates that degradation at the anode, manifested as slippage, plays a more critical role in disrupting full-cell electrochemical balance than cathode-side changes.

To further clarify the dominant contribution of the anode to full-cell capacity loss at elevated cut-off voltages, post-mortem half-cell electrochemical analysis was conducted. After cycling, the full-cells were disassembled, and the cathode/anode electrodes were reassembled into Li-metal half-cells, respectively, to compare their galvanostatic curves (Figure 1(d)). To minimize kinetic effects, a low current of 0.05 C was applied, and to simulate the charging situation in full-cell cycling, the system was designed to accommodate charging at a voltage equivalent to the full-cell charging by adding the reduction potential of the graphite anode (0.05 V *vs*. Li/Li^+^). Figures 1(e,f) show the half-cell galvanostatic profiles of SC-NCM6 cathodes before and after long-term evaluation of 4.35 V and 4.40 V full-cells, respectively. The capacity measured in the fresh half-cell at 4.35 V SC-NCM6 was 202.5 mAh/g, with a capacity decrease of 20.2 mAh/g (Figure 1(e), (c)) occurring after full-cell cycling. In the 4.40 V SC-NCM6, which had an initial capacity of 205.7 mAh/g, a cathode capacity decrease (Figure 1(f), (c)) of 28.0 mAh/g occurred after 600 charge/discharge cycles. In terms of capacity retention, 4.35 V SC-NCM6 and 4.40 V SC-NCM6 showed 90% and 86%, respectively, confirming that the cathode degraded slightly more when operated at 4.40 V. In contrast, for the anode, the reversible capacity decrease (Figure 1(g,h), (a)) was about 59 mAh/g under 4.35 V cycling conditions, whereas at 4.40 V, it was approximately twice as much at 110 mAh/g. Given that commercial graphite typically delivers an initial reversible capacity of approximately 350 mAh/g, this indicates that nearly one-third of the capacity becomes inaccessible. These findings confirm that the dominant source of capacity loss under high-voltage operation is not cathode degradation, but severe anode degradation caused by lithium inventory imbalance.

### SC-NCM6 cathode degradation behavior during high-voltage cycling

**Figure 2.**
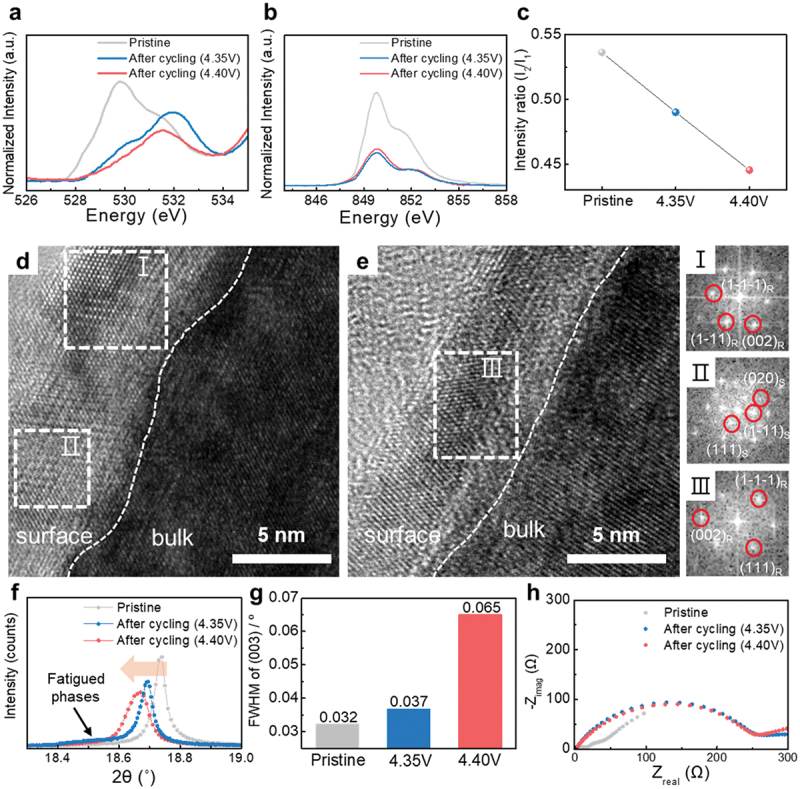
Post-mortem analyses of cycled SC-NCM6 cathodes. (a) Soft XAS O K-edge spectra of the cycled SC-NCM6 cathode. (b) Soft XAS Ni L_3_-edge spectra and (c) relative intensity ratio between low energy peak to high energy peak (Ni L_3 low_/L_3 high_). HR-TEM images of the cycled electrodes: (d) Cycled at 4.35 V, and (e) cycled at 4.40 V in full-cell cycles. FFT images of the selected area (I, II, III) are labeled by ‘I, II, III’. R represents a peak from rocksalt phase; S represents a peak from spinel phase. (f) XRD patterns of Bragg peak (003) from the cycled SC-NCM6. (g) Integrated O K-pre edge peak areas and FWHM of the (003). (h) The EIS profiles of the cycled electrodes.

In an ideal full cell with an optimized N/P ratio, increasing the upper cut-off voltage extracts additional capacity from the cathode, while the anode operates within a relatively stable potential range where lithium plating is avoided. Under such conditions, degradation is typically expected to originate from the cathode side. However, the observed acceleration of anode degradation at high voltage indicates that the anode was unexpectedly impacted, likely due to cross-talk effects originating from the cathode rather than direct electrochemical stress on the anode itself [27,36,37]. This suggests that degradation at the anode is not simply a consequence of high-voltage cycling, but a cathode-induced phenomenon that disrupts the expected electrode stability balance. To investigate the degradation behavior of the SC-NCM6 cathode under high-voltage conditions, TEM and synchrotron-based X-ray analyses – including soft-XAS and HRPD – were conducted to probe structural changes at both the surface and bulk levels (Figure 2).

The breakdown of the layered structure accompanied by oxygen release at the SC-NCM6 surface was confirmed through soft-XAS surface analysis. Using synchrotron-soft XAS in TEY mode with a beam size of 300 × 600 μm^2^ (Figure 2(a)), we observed average changes in the chemical bonds around oxygen ions and the electronic structure of transition metal ions in regions approximately 3 nm deep from the SC-NCM6 surface. In the O K-edge spectra, the pre-edge region appearing at 527–534 eV represents O 1s transitions to hybridized TM_3d_-O_2p_ states [38]. After full-cell cycling, the areal intensity of the pre-edge in the O K-edge decreased, and the signal at 531–532 eV corresponding to the TM-O hybridized state of disordered spinel or rock-salt increased relative to the intensity near 528–530 eV, which corresponds to the TM-O hybridized state of the layered phase (Figure 2(a)) [38]. Notably, this trend was observed to be more severe in the 4.40 V electrode, indicating that lattice oxygen evolution at the surface occurred more intensely during high-voltage operation.

The reduction of Ni ions near the surface compared to their initial state after cycling was evidenced through Ni L_3_-edge TEY mode analysis (Figure 2(b)). The oxidation state of Ni ions affects their electronic configuration and ligand-field environment, altering multiplet structures and thus the Ni L_3_-edge spectral shape; the smaller the relative ratio of the shoulder peak intensity (I_2_) near 852 eV compared to the first peak intensity (I_1_) around 850 eV in the L_3_-edge, the closer the oxidation state of Ni ions is to ‘2+’ rather than ‘3+’ [38,39]. Indeed, the relative intensity (I_2_/I_1_) within the Ni L_3_-edge peak decreased after full-cell cycling, and this value was smaller in the 4.40 V cycled cathode compared to the 4.35 V cycled cathode (Figure 2(c)). These findings, consistent with the O K-edge results, further indicate enhanced oxygen release at the cathode surface with increasing cut-off voltage.

Accelerated surface phase transitions and structural collapse at high voltages in SC-NCM6 were confirmed by HR-TEM analysis. Before cycling, the SC-NCM6 surface was confirmed to have a layered structure (Figure S5). After full-cell cycling, the surface commonly existed not as a layered phase but as spinel (*Fd3m*) or cubic structure (*Fm-3 m*). A distinctive feature was that in the 4.35 V cathode surface, mixed regions of cubic (I) and spinel (II) structures were frequently observed (Figure 2(d)), while in the 4.40 V case, only cubic regions (III) were observed (Figure 2(e)), which has been reported in other ternary layered oxide [40,41]. These surface structural changes might originated from H-transfer reactions facilitated by the Ni lattice, transferring protons from electrolyte solvent molecules to the cathode O lattice, which potentially accelerating transition metal dissolution [29]. Consequently, dissolved transition metals can migrate to and deposit on the anode, promoting surface side reactions. Thus, the observed cathode surface degradation likely contributed significantly to the anode degradation shown in Figure 1(g,h) [26].

The acceleration of bulk degradation as well as surface deterioration during high-voltage cycling was observed through HRPD. Among all Bragg peaks, (00 l) reflections showed the most significant broadening, with the high-intensity (003) peak used to track structural evolution in SC-NCM6 after cycling (Figure S6). After full-cell cycling, the (003) peak shifted to the left compared to the pristine cathode in all cycled cathodes, with a greater leftward shift at higher charging voltages. This suggests that despite fully discharging the cathode, internal lithium ions may not have been completely filled due to increased charge transfer resulting from surface degradation. Additionally, examining the FWHM change of the (003) peak, the 4.35 V cycled cathode showed approximately a 12% increase compared to the pristine cathode, while the 4.40 V cycled cathode showed a 103% increase (Figure 2(g)). From the SEM images of the cross-section of cathode materials after full-cell test in Figure S7, more internal cracks were observed in the 4.40 V cathode compared to the 4.35 V cathode. From the viewpoint of crystallite size, internal cracking of the single-crystal cathode material may have generated smaller crystallites, contributing to the observed peak broadening. Furthermore, a fatigue phase appeared at 2θ = 18.40–18.55 ° left of the cycled cathode’s (003) peak (Figure S8). Previous studies reported that high-voltage cycling of single-crystal NCM causes chemical inhomogeneity and particle deformation due to repeated lattice expansion and contraction [42]. Higher voltages promote surface oxygen release and accelerate bulk degradation [24]. This irreversible structural deformation during cycling likely contributed to the observed peak broadening, as confirmed by the correlation between integrated intensity of O K pre-edge and FWHM of (003) for SC-NCM6 (Figure S9).

To assess the impact of cathode surface oxygen release and reconstruction on electrochemical activity under high-voltage operation, electrochemical impedance spectroscopy (EIS) analysis was performed. The resistance (R = R_SEI_ + R_charge transfer_) measured by EIS of SC-NCM6 after 4.35 V and 4.40 V cycling increased slightly at the higher voltage, with values of 257 Ω and 264 Ω, respectively (Figure 2(h)). This minor increase can be attributed to more severe oxygen release and surface reconstruction at the cathode surface under 4.40 V conditions, which is consistent with the greater reduction in reversible cathode capacity observed in Figure 1(e–f). Notably, however, the resistance ratio (R_4.40 V_/R_4.35 V_) was only 1.03, whereas the relative increase in anode slippage capacity (Q_slippage,4.40 V_/Q_slippage,4.35 V_) in the full-cell (Figure 1(b–c)) was substantially higher at 1.18. This indicates that although cathode surface degradation contributes to increased interfacial resistance, its effect on overall full-cell performance is limited compared to the more pronounced impact of anode degradation under high-voltage conditions.

### Cross-talk-mediated surface degradation of graphite anode

Cross-talk phenomena originating from the cathode – specifically transition metal dissolution and oxygen release – have been reported to accelerate anode degradation in full-cell systems [26,27,29]. For instance, deposited transition metals on the anode surface can enhance side reactions with the electrolyte, promoting thicker SEI formation, or catalyze hydrogen gas evolution, ultimately accelerating Li inventory loss in full-cells [28,29]. Although graphite anodes are generally considered structurally and electrochemically stable and cyclable in EC/EMC/DEC-based electrolytes (Figure S10) [43,44,45], the degradation observed in SC-NCM6/graphite full-cells suggests that their interfacial stability can be compromised by cross-talk-induced SEI modifications. To explore these effects in detail, a comprehensive surface analysis of the graphite anode was conducted using SEM, TOF-SIMS, and XPS [27,45].

**Figure 3.**
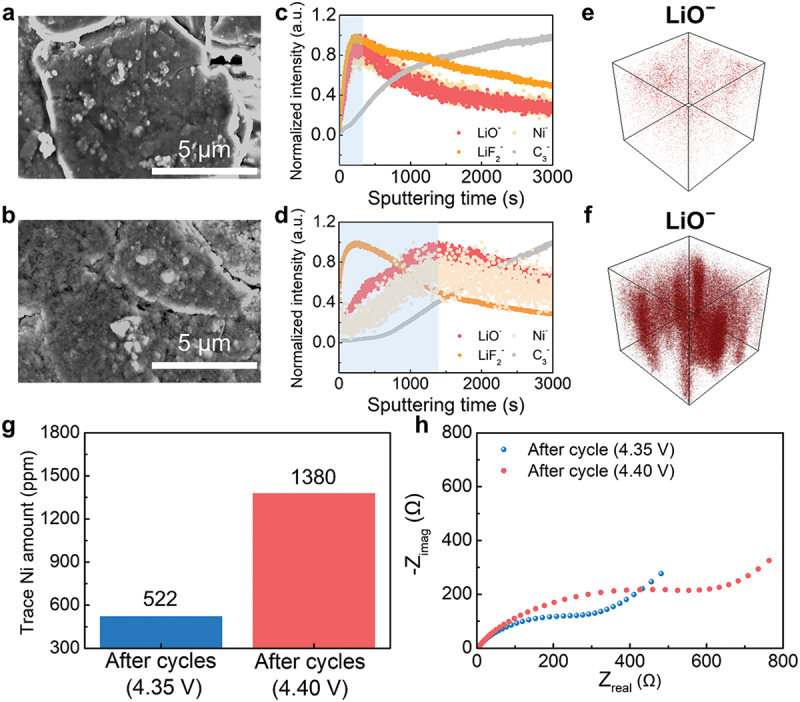
Post-mortem analyses of cycled graphite anodes. SEM images of graphite electrodes operated at (a) 4.35 V and (b) 4.40 V cycling. Normalized TOF-SIMS depth profiles and corresponding 3D LiO^−^ distribution plots for graphite electrodes cycled at (c, e) 4.35 V and (d, f) 4.40 V. All spectra are normalized to their respective maximum intensities. (g) ICP-OES analysis showing trace Ni content in cycled separators. (h) EIS profiles of the cycled electrodes.

SEM imaging revealed that SEI byproducts formed on both 4.35 V and 4.40 V cycled anodes (Figure 3(a,b)). In the 4.35 V case, they were discretely deposited on individual particles, while at 4.40 V, they formed a continuous and thicker coating (Figure S11). The absence of needle-like byproducts in both cycled anodes, combined with the previous electrochemical analysis results, provides indirect evidence that graphite anode degradation at high voltage stemmed from SEI layer modifications rather than Li-plating on the surface [46].

Graphite anode surface degradation was found to intensify with increasing charging voltage, primarily due to transition metal dissolution. This behavior was examined in detail using TOF-SIMS depth profiling to characterize SEI layer evolution on the graphite surface (Figure 3(c,d)). According to prior studies, the SEI layer typically consists of inorganic species near the surface and organic byproducts at greater depths [28,29]. To capture this structural profile and evaluate transition metal dissolution effects, we monitored signals from Li-based components (LiF_2_^−^, LiO^−^), graphite (C_3_^−^), and transition metal species (Ni^−^, NiF_2_^−^, MnF_3_^−^, CoF_3_^−^), normalized to their maximum intensities across the measured depth range [27]. The depth profiles of LiF_2_^−^ – an indicator of inorganic SEI species – exhibited similar spatial distributions regardless of charging voltage. This suggests that LiPF_6_ salt decomposition, which generates such inorganic species, is relatively insensitive to voltage variation under these conditions [45]. In contrast, LiO^−^ signals – representing organic components such as Li*_x_*O and Li_2_CO_3_ – penetrated deeper into the SEI and showed greater intensity at 4.40 V than at 4.35 V (Figure 3(e,f)). This observation indicates that high-voltage operation accelerates the formation and thickening of organic-rich SEI layers.

Notably, the shaded regions in Figure 3(c,d) reveal strong overlapping LiO^−^ and Ni^−^ profiles, implying a close relationship between transition metal dissolution and organic SEI formation. Such alignment suggests that Ni ions dissolved from the cathode migrate toward the anode and locally catalyze side reactions, thereby enhancing the growth of electrochemically inactive organic species within the SEI [28]. These findings point to a cross-talk mechanism where cathode-derived Ni facilitates SEI thickening, ultimately impairing anode surface properties under high-voltage conditions.

This correlation was further validated by surface-sensitive compositional analyses. XPS Ni 2p spectra showed stronger Ni signals in the 4.40 V-cycled graphite anode than in the 4.35 V case (Figure S12), and ICP-OES analysis of the separator confirmed that Ni content at 4.40 V was more than twice that at 4.35 V (Figure 3(g)). Consistently, EIS measurements revealed a significant increase in anode resistance, with values of 581 Ω at 4.40 V and 292 Ω at 4.35 V (Figure 3(h)). These results establish a strong relationship between transition metal accumulation and interfacial impedance increase. Notably, the resistance ratio of the anode (R_4.40 V_/R_4.35 V_) was substantially higher than that of the cathode (1.03), paralleling the capacity loss trends observed in post-mortem half-cell tests – where the anode showed a 110 mAh/g loss, nearly four times that of the cathode (28 mAh/g). Collectively, these findings confirm that reversible capacity reduction in high-voltage full-cells is primarily driven by anode-side degradation, which originates from cathode-induced cross-talk and results in progressive cell balance mismatch.

## Conclusion

Single-crystal mid-Ni layered oxide (SC mid-Ni NCM) cathodes have emerged as promising candidates for EV applications, offering suppressed surface side reactions and superior thermal stability compared to high-Ni counterparts. However, high-voltage operation (≥4.35 V *vs*. graphite) is required to overcome the limited energy density inherent to their moderate Ni content. Despite growing interest, the full-cell degradation mechanisms of systems incorporating this material under such high-voltage conditions remain poorly understood.

**Figure 4.**
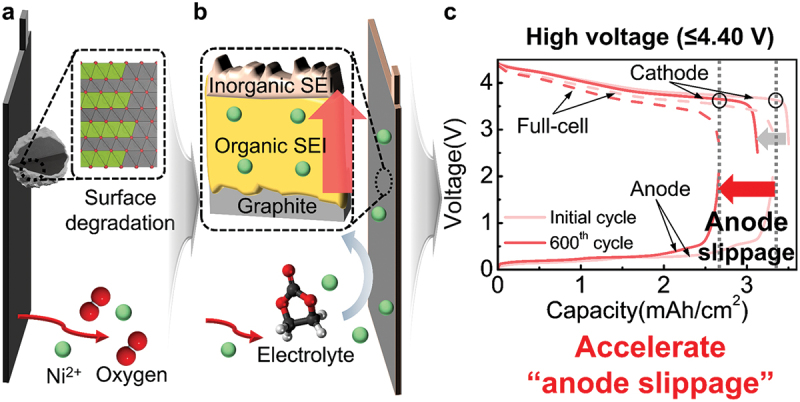
Schematic illustration of SC-NCM6/graphite degradation mechanism operating at high voltage: (a) Cathode surface degradation accompanied by oxygen loss and nickel ion dissolution. (b) Anode surface degradation induced by dissolved Ni ions from the cathode. (c) Occurrence of anode slippage and full cell capacity loss. Red and grey arrows indicate the amount of the capacity induced by the anode slippage and cathode degradation each.

In this study, we reveal that full-cell capacity loss is predominantly governed by anode degradation – specifically anode electrode slippage – induced by cross-talk from cathode Ni dissolution. During early cycling stages, high-voltage conditions accelerate phase transitions at the cathode surface (from layered to rock-salt), along with lattice oxygen release and transition metal dissolution, particularly of Ni (Figure 4(a)). Dissolved Ni ions migrate through the electrolyte and deposit on the anode surface, where they promote parasitic side reactions and the continuous growth of organic-rich SEI layers (Figure 4(b)). This thickened SEI layer significantly increases charge transfer resistance at the anode, reducing its electrochemical activity and leading to a progressive mismatch in electrode capacity. As a result, anode potential slippage occurs, ultimately disrupting cell balance and dominating reversible capacity loss during long-term cycling (Figure 4(c)).

While previous studies on high-voltage NCM/graphite full cells have primarily focused on cathode degradation or cross-talk in general, our differential voltage analysis and post-mortem evaluation demonstrate that anode-side degradation plays a more decisive role than cathode deterioration. Notably, the sharp rise in interfacial resistance at the anode under high-voltage operation, rather than cathode degradation alone, emerges as the principal factor in full-cell failure. This highlights the importance of addressing anode slippage and preserving electrode balance in the design of stable, high-voltage mid-Ni full-cell systems. This work not only clarifies the dominant role of anode degradation in high-voltage mid-Ni full cells but also provides a framework for future strategies aimed at mitigating cross-talk and preserving long-term cell balance.

## Supplementary Material

Supplemental Material
