# Supplementary material for: Understanding cross-talk–induced anode slippage in high-voltage mid-Ni NCM/graphite full cells
Source: Sci Technol Adv Mater. 2025 May 16;26(1):2502324. doi: 10.1080/14686996.2025.2502324 (PMC12160330; doi:10.1080/14686996.2025.2502324)
Supplement: Supplemental Material [file TSTA_A_2502324_SM6416.docx]

**Supporting Information**

**Understanding cross-talk–induced anode slippage in high-voltage mid-Ni NCM/graphite full cells**

Seungjae Suk^a^, Namgyu Yoo^b^, Youngsu Lee^b^, Jaesub Kwon^a^, Heeju Ahn^c^, Seungsu Yoo^c^, Jaewoon Lee^d^, Haneul Kim^c^, Joongho Bae^c^, Jongwoo Kim^c^, Chiho Jo^c^, Young-Tae Kim^a^ and Kyu-Young Park^a,b*^

^a^Department of Materials Science and Engineering, Pohang University of Science and Technology (POSTECH), Pohang 37673, Republic of Korea.
 ^b^Graduate Institute of Ferrous & Eco Materials Technology (GIFT), Pohang University of Science and Technology University, Pohang 37666, Republic of Korea
^c^LG Energy Solution, Research Park, Daejeon, 34122, Republic of Korea
^d^Department of chemical engineering and Materials Science, University of Minnesota–twin cities, Minneapolis, MN 55455, USA

^*^Dr. Kyu-Young Park,

Assistant Professor of Graduate Institute of Ferrous & Eco Materials Technology

Department of Materials Science and Engineering

Address: 40, Jigok-ro 212beon-gil, Nam-gu, Pohang-si, Gyeongsangbuk-do, Republic of Korea (Zip code: 37667); Mail: [kypark0922@postech.ac.kr](mailto:kypark0922@postech.ac.kr)

ORCID: <https://orcid.org/0000-0001-9560-8526>


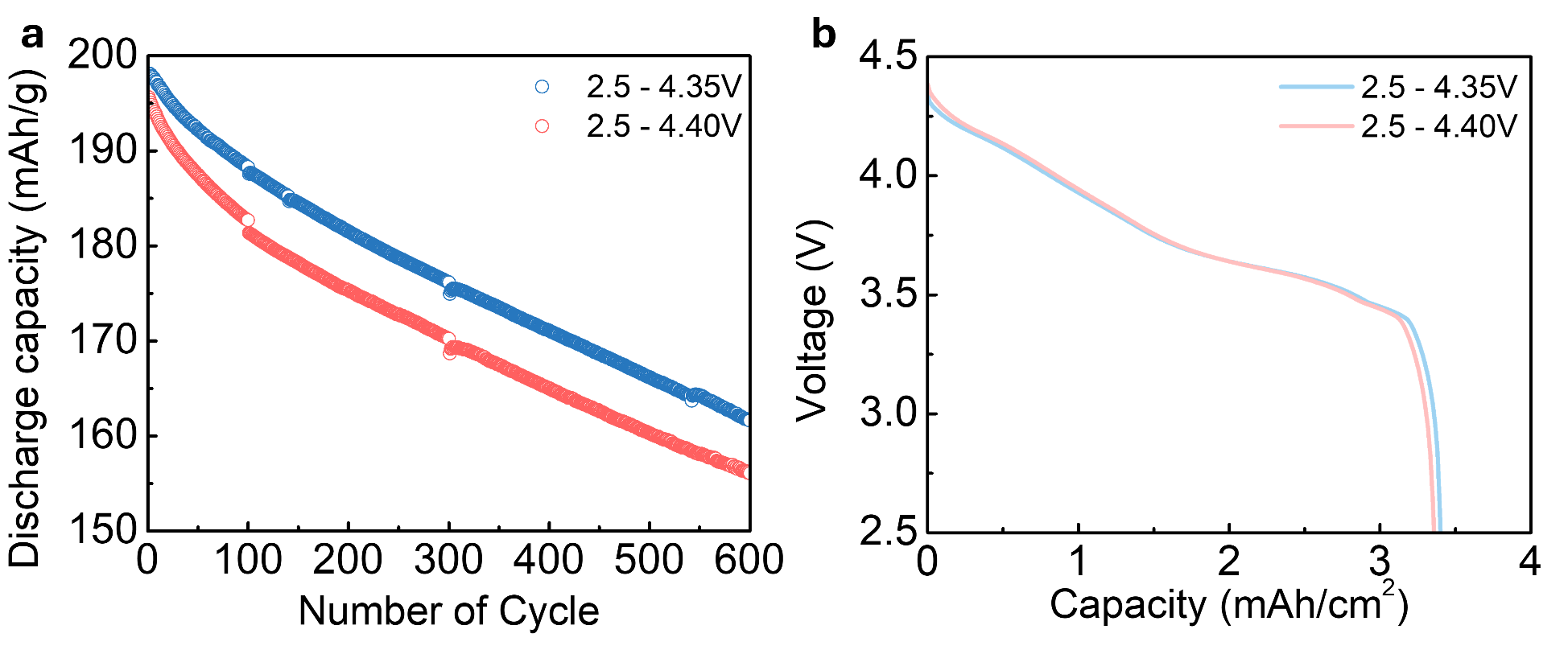


Figure S1. (a)Discharge capacity for the pouch cells cycled at 0.33C between 2.5-4.35 V and 2.5-4.40 V at 45 ℃. (b)Discharge capacity-voltage curve of 1^st^ cycle for each full-cell. Surprisingly, despite the elevated charging voltage of 4.40 V compared to 4.35 V, the full-cell exhibits a reduced initial discharge capacity by 2.5 mAh/g (=0.05 mAh/cm²). A detailed explanation of this phenomenon is provided in Figure S3.


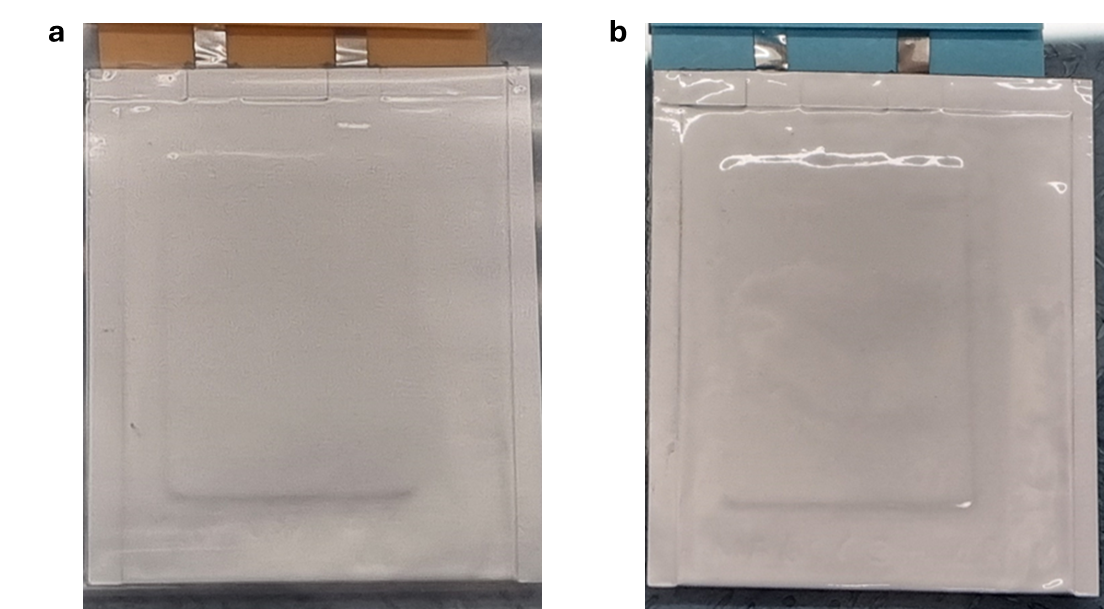


Figure S2. (a) 4.35 V and (b) 4.40 V cycled full-cell appearance after 600 cycles. The full-cell cycled at 4.40 V exhibited slightly greater internal gas accumulation compared to the one cycled at 4.35 V. This difference can be explained by those combined mechanisms: higher voltage operation promoting oxygen release from the cathode surface^[4]^ and electrolyte decomposition.


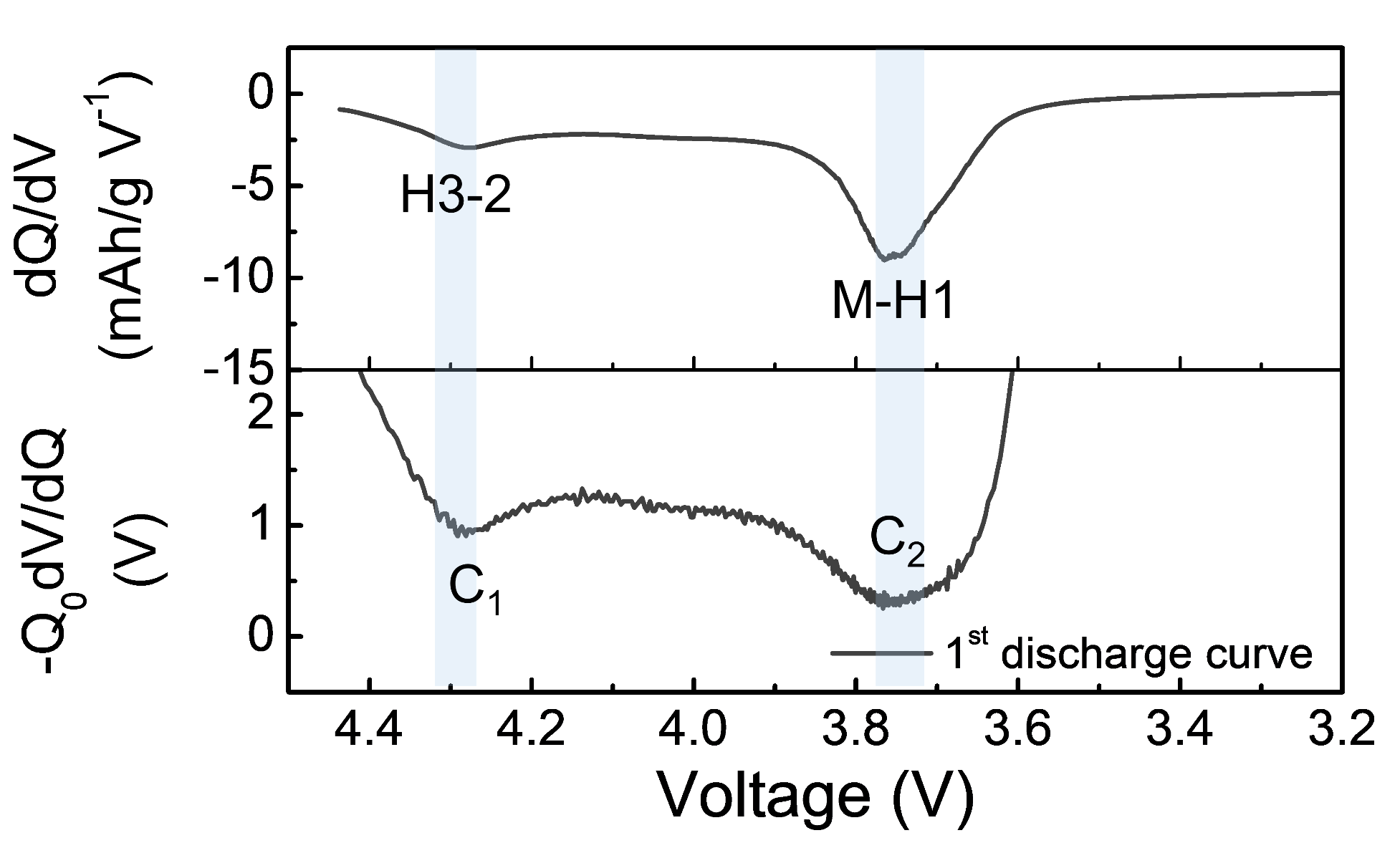


Figure S3. The dQ/dV and –Q_0_dV/dQ curves from SC-NCM6 cathode half-cell operated at 2.5-4.40 V (*vs.* Li/Li^+^) with 0.3C at 45 ℃. In the -Q_0_dV/dQ profile, the local minima at ~4.28 V and ~3.76V correspond to two-phase reactions occurring during lithium insertion into SC-NCM6. The phase transition processes are labelled based on previous literature.^[1]^ The 4.28 V minimum is attributed to the H3 to H2 phase transition, while the 3.76 V minimum indicates the coexistence of monoclinic and hexagonal phases.


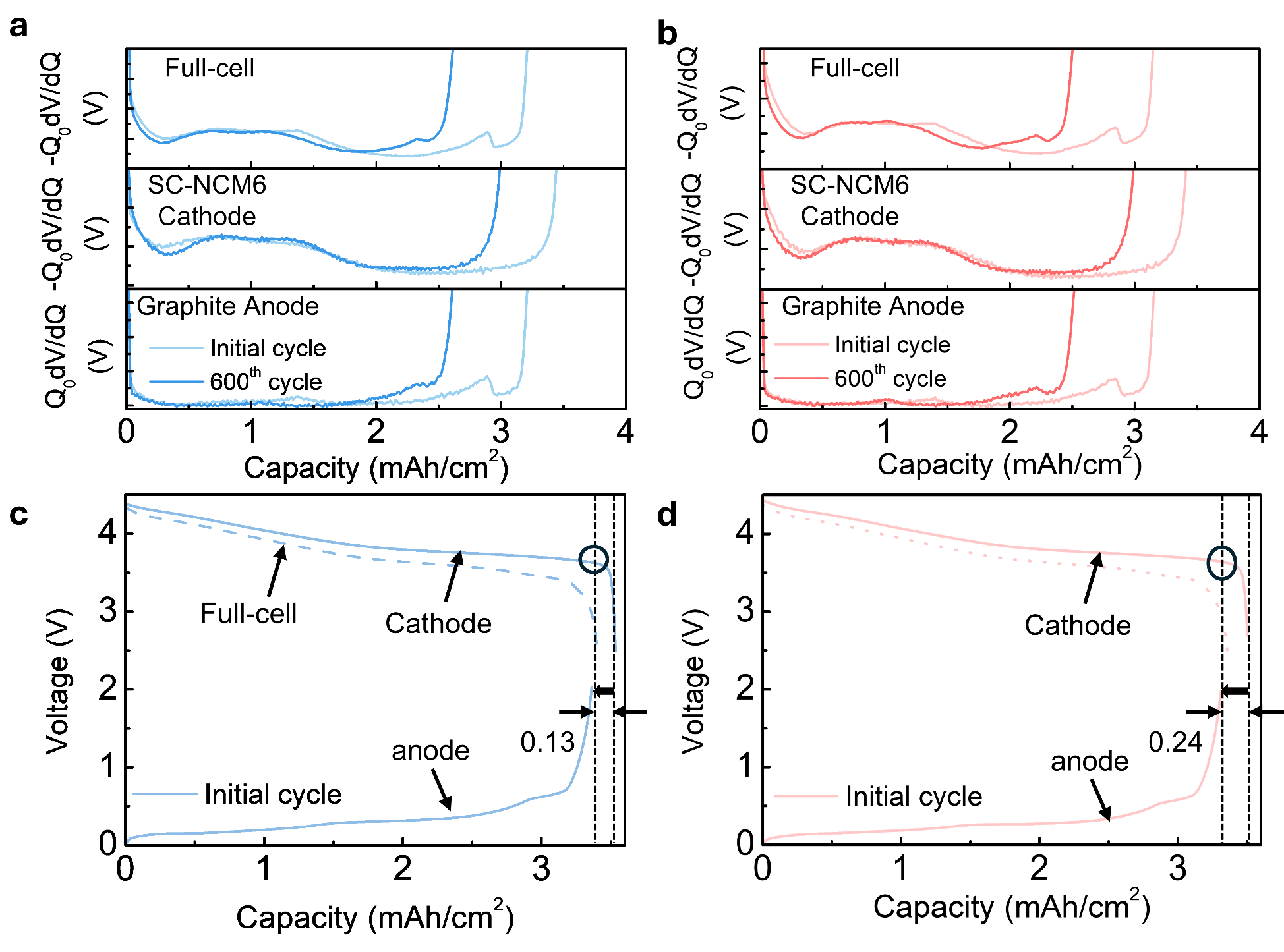


Figure S4. The Q_0_dV/dQ curves of the full-cells, SC-NCM6 cathodes and graphite anodes operated at (a) 4.35 V and (b) 4.40 V. The curves for SC-NCM6 were obtained from half-cell experimental data. The anode curves were theoretically derived by subtracting the SC-NCM6 half-cell data from the full-cell data. The galvanostatic curves of the full-cells, cathodes and anodes operated at (c) 4.35 V and (d) 4.40 V based on the full-cell voltage. The black arrows indicate the amount of relative anode slippage after formation.^[3]^ Compared to 4.35 V, slippage occurs 0.11 mAh/cm^2^ more at 4.40 V, which leads to lower initial discharge capacity.


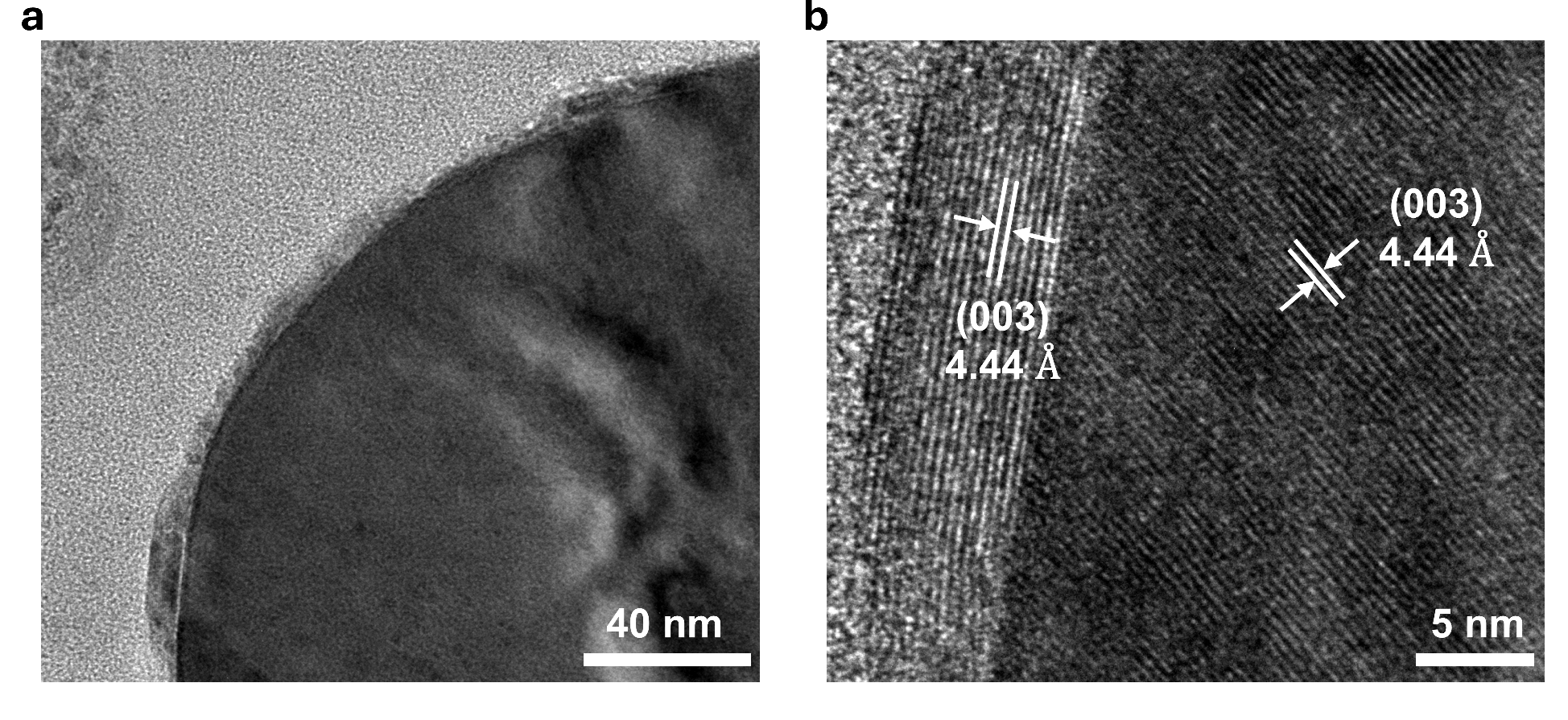


Figure S5. HR-TEM images of the pristine SC-NCM6. (a) HR-TEM visualization at low magnification (b) High-magnification image and the d-spacing value of the nearby surface is 4.44 Å, which indicates the (003) plane of the layered structure (R3̅m).


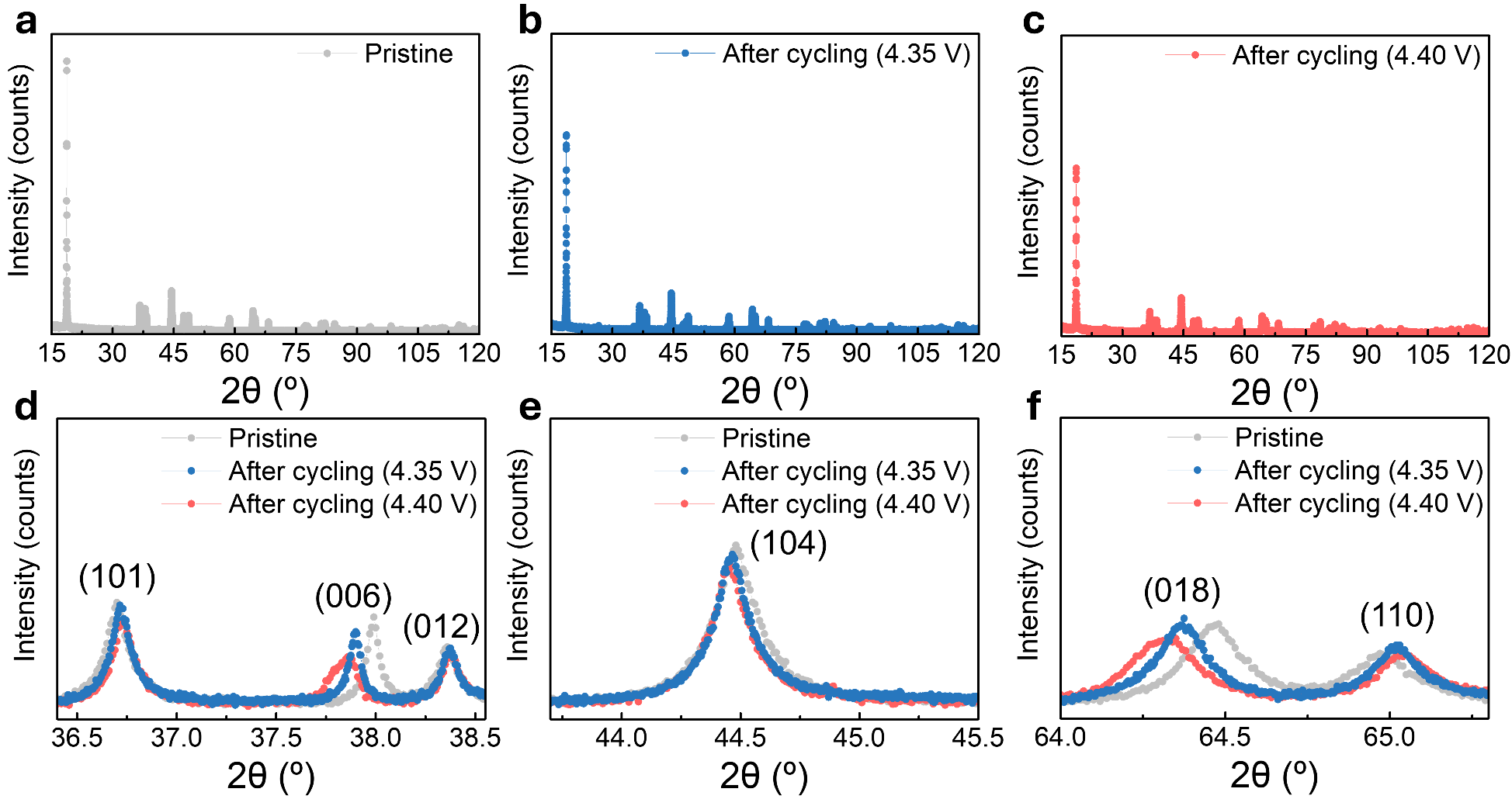
 Figure S6. HRPD patterns of (a) pristine SC-NCM6 and electrodes after cycling at (b) 4.35 V and (c) 4.40 V (vs graphite). XRD patterns showing (d) (101), (006), and (012) reflections; (e) (104) reflection; and (f) (018) and (110) reflections.


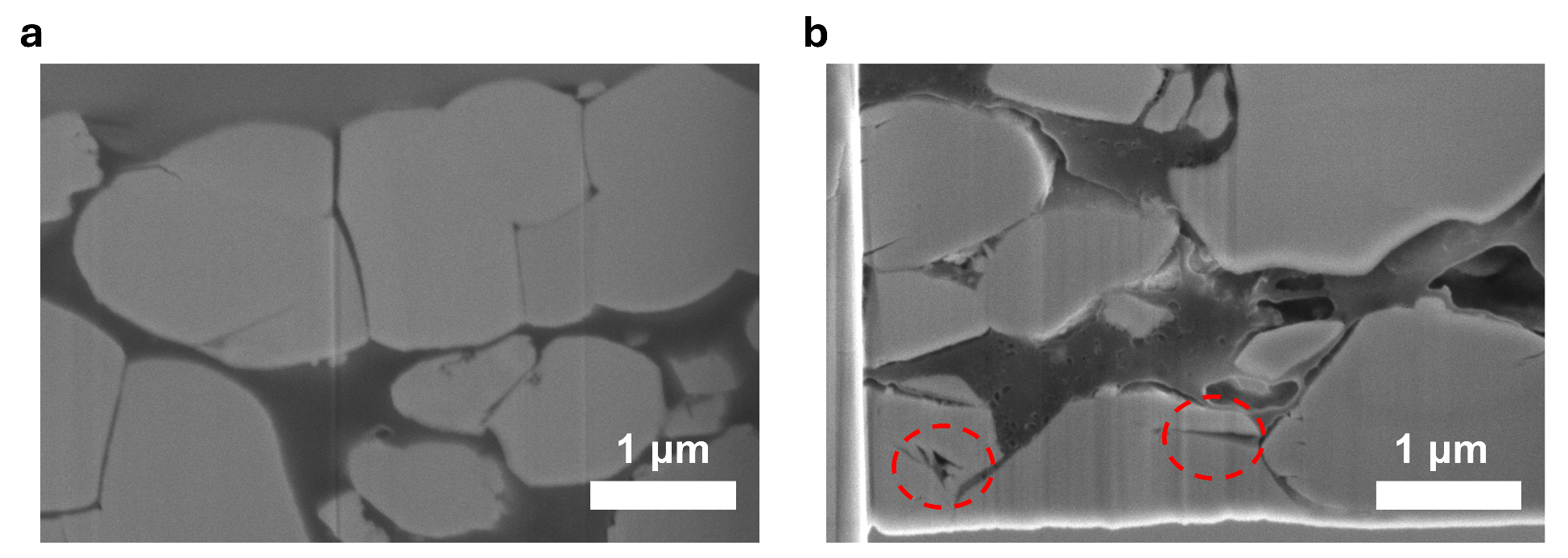


Figure S7. Cross-sectional SEM image of the cycled (a) 4.35 V and (b) 4.40 V SC-NCM6. The red circles indicate the microcracks.


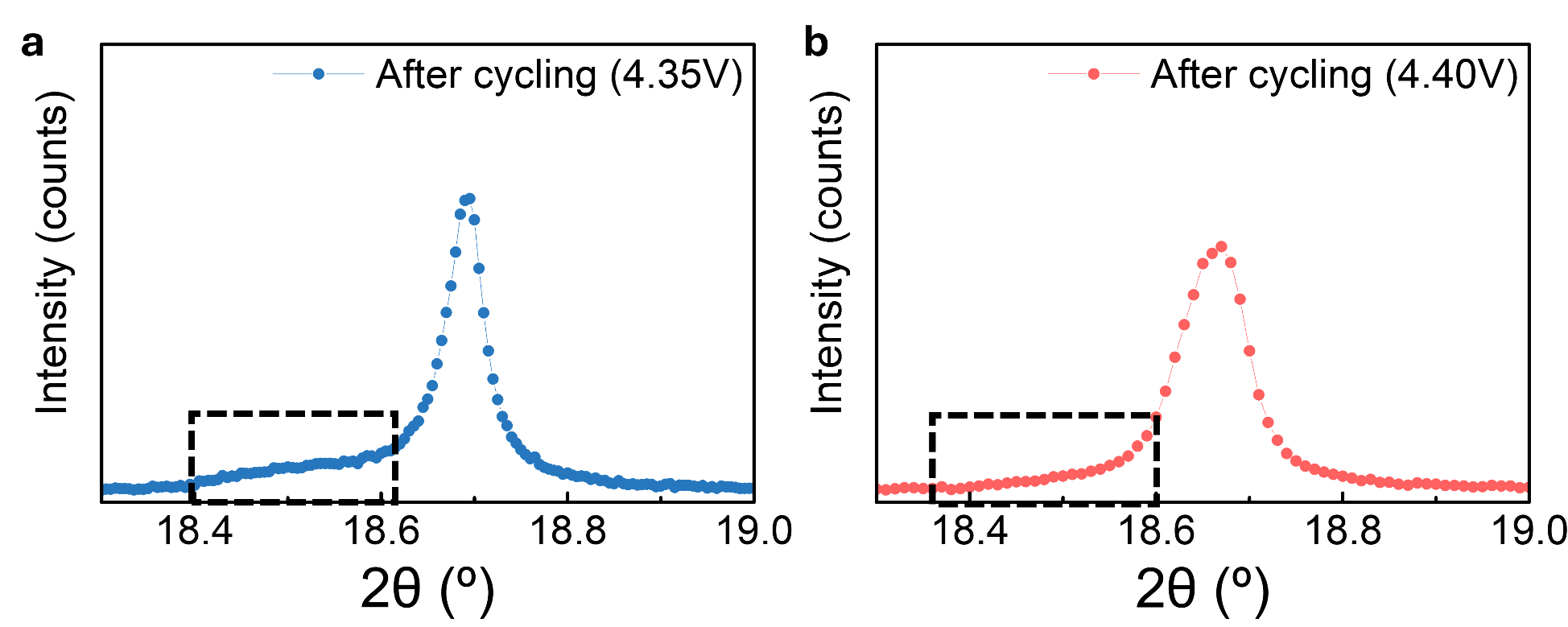


Figure S8. Fatigued phases near the (003) Bragg peak in SC-NCM6 cycled at (a) 4.35 V and (b) 4.40 V. Dotted square box indicates the existence of fatigued phases.

Figure S9. Trend of integrated intensity of O K pre-edge and FWHM of (003) for pristine, 4.35V and 4.40V cycled SC-NCM6. As the areal intensity of O K pre-edge decreases, the FWHM of (003) increases, indicating that surface oxygen release could accelerate bulk degradation.


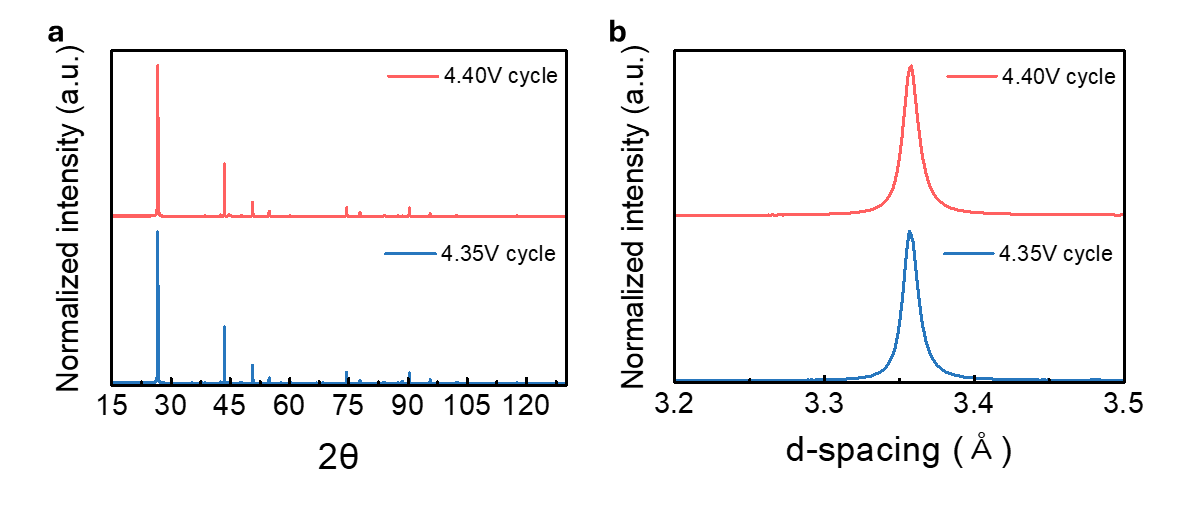


Figure S10. (a) HRPD patterns of graphite anode electrodes after cycling at 4.35 V and 4.40 V (vs graphite). (b) Comparison of d-spacing of the (002) plane for the cycled anode. Analysis of the graphite anodes revealed that the FWHM of the (002) peak measured 0.12075 ˚ and 0.12471 ˚ for samples cycled at 4.35 V and 4.40 V (*vs.* Li/Li+), respectively. The d-spacing values for the (002) plane remained constant at 3.356 Å across both voltage conditions. These findings suggest negligible structural changes in the graphite anode when the upper charging voltage was increased.


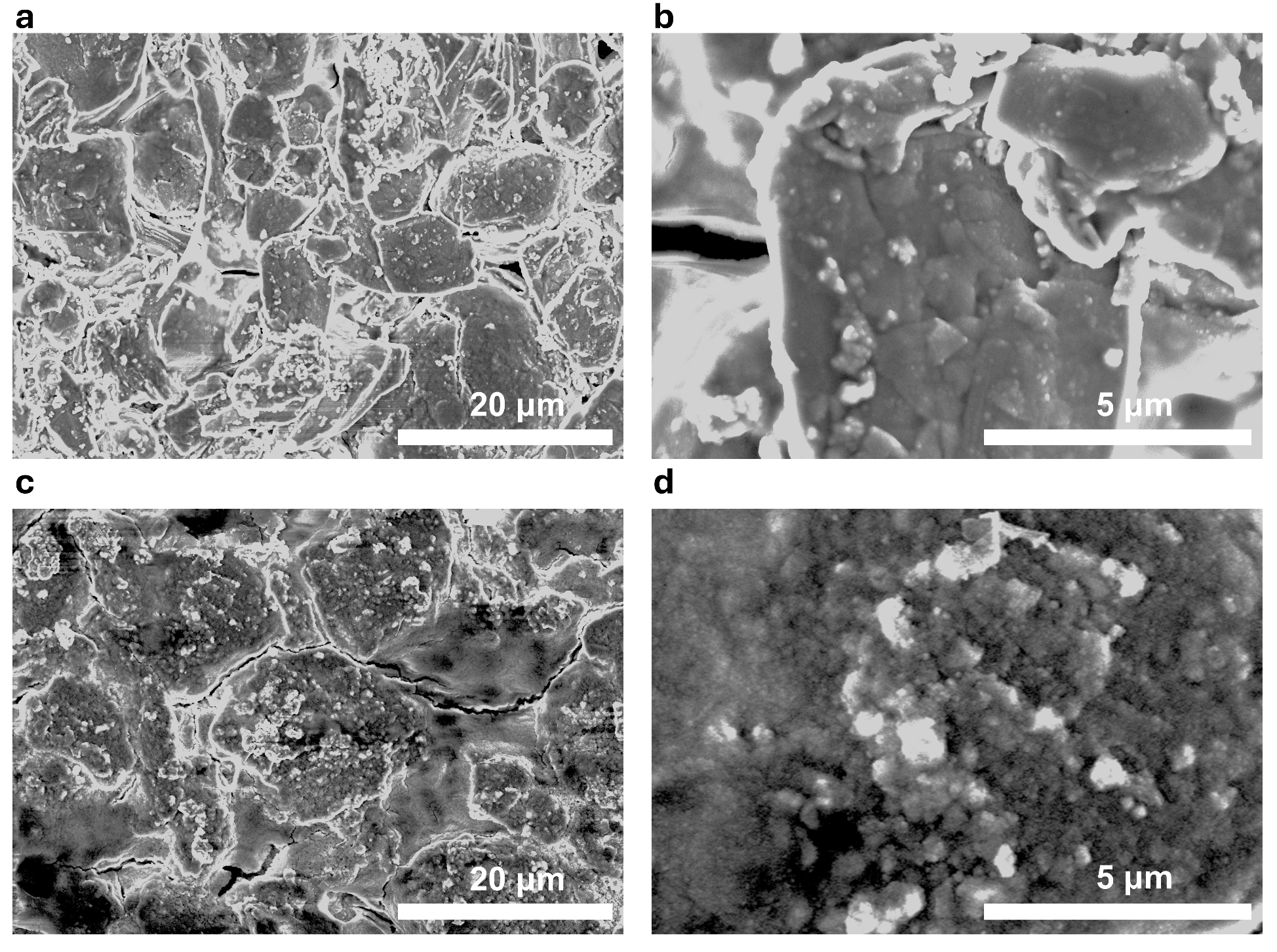


Figure S11. SEM images of graphite electrodes operated at (a,b) 4.35 V and (c,d) 4.40 V cycling.


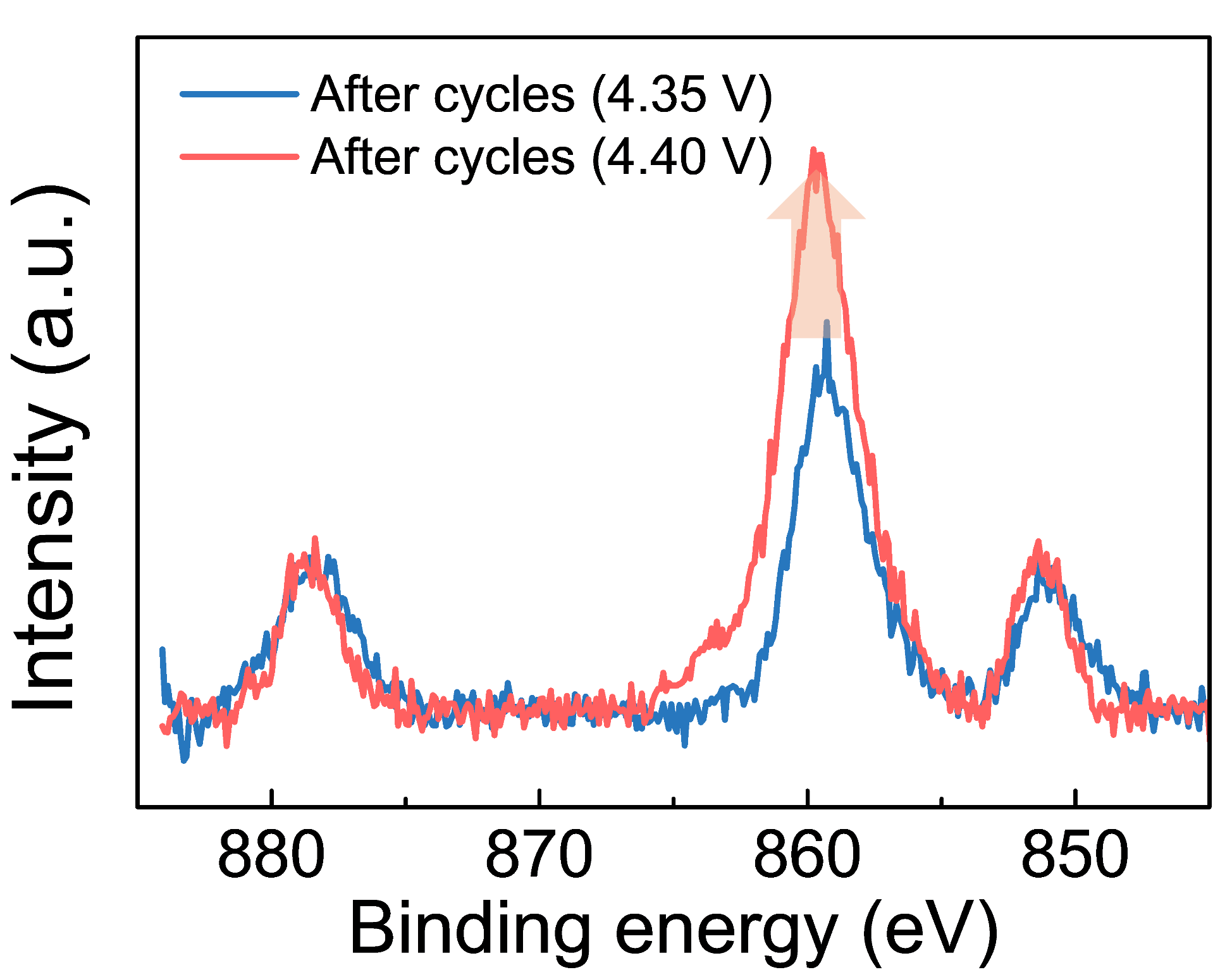


Figure S12. XPS spectra of the cycled graphite anodes. All spectra were obtained after surface etching to eliminate other irrelevant impurities.

Reference

[1] Liu T, Yu L, Liu J, et al. Understanding Co roles towards developing Co-free Ni-rich cathodes for rechargeable batteries. Nat Energy. 2021;6:277–286. doi: 10.1038/s41560-021-00776-y

[2] Ryu HH, Yoon CS, Sun YK, et al. Capacity Fading of Ni-Rich Li[Ni*_x_*Co*_y_*Mn_1–_*_x_*_–_*_y_*]O₂ (0.6 ≤ *x* ≤ 0.95) Cathodes for High-Energy-Density Lithium-Ion Batteries: Bulk or Surface Degradation? Chem. Mater. 2018;30:1155–1163. doi: 10.1021/acs.chemmater.7b05269

[3] Gauthier R, Louli, A, Dahn, JR, et al. How do Depth of Discharge, C-rate and Calendar Age Affect Capacity Retention, Impedance Growth, the Electrodes, and the Electrolyte in Li-Ion Cells? J Electrochem Soc. 2022;169(2) doi: 10.1149/1945-7111/ac4b82

[4] Park KY, Zhu YZ, Torres-Castanedo CG, et al. Elucidating and mitigating high-voltage degradation cascades in cobalt-free LiNiO_2_ lithium-ion battery cathodes. Adv Mater. 2021;34(3). doi: 10.1002/adma.202106402
